# Correlação Angiotomográfica-Eletrocardiográfica na Síndrome de Wellens

**DOI:** 10.36660/abc.20200200

**Published:** 2021-02-19

**Authors:** Eduardo Kaiser Ururahy Nunes Fonseca, Nevelton Heringer, Marcelo L. Montemor, Luiz Francisco Rodrigues de Ávila, Carlos Eduardo Rochitte

**Affiliations:** 1 Universidade de São PauloFaculdade de MedicinaHospital das ClínicasSão PauloSPBrasilUniversidade de São Paulo Faculdade de Medicina Hospital das Clínicas Instituto do Coração, São Paulo, SP - Brasil

**Keywords:** Eletrocardiografia, Vasos Coronários, Angiografia por Tomografia Computadorizada, Infarto do miocárdio, Angiografia coronária

A Síndrome de Wellens,^1^ também conhecida como “Síndrome da onda T da Coronária Descendente Anterior” foi descrita em 1982 pelo Dr. Henrick Joan Joost (Hein) Wellens, médico holandês que também contribuiu com a caracterização do mecanismo de reentrada na síndrome de Wolf Parkinson White.

Descrita originalmente durante a admissão hospitalar (60% à admissão e 40% no seguimento) de pacientes apresentando angina instável, foi caracterizada pela ocorrência de 2 padrões eletrocardiográficos, padrão A em 25% dos pacientes e B em 75% dos pacientes.

No padrão A nota-se a ocorrência de onda T bifásica nas derivações V2 e V3 podendo ser encontrada de V1 a V6 e no padrão B nota-se onda T invertida e simétrica em V2 e V3, ambos padrões ocorrendo sem a associação de ondas Q ou complexos QS patológicos, com progressão normal da onda R e sem evidência de hipertrofia ventricular.

Tais achados eletrocardiográficos são pouco sensíveis (69%), porém altamente específicos (89%)^2^de doença obstrutiva importante do segmento proximal da artéria coronária descendente anterior que, se não abordada de forma adequada, pode determinar infarto anterior extenso e alto risco de mortalidade.

Sendo assim, na vigência de achados eletrocardiográficos da Síndrome de Wellens é desencorajada a realização de testes provocativos de isquemia.^3^

**Figura 1:**
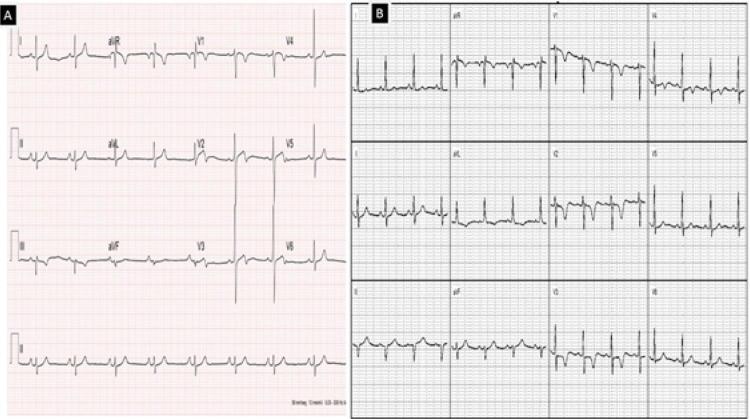
Imagens dos ECGs de ambos os pacientes, evidenciando os padrões da síndrome de Wellens (Paciente 1 - A / Paciente 2 - B).

**Figura 2:**
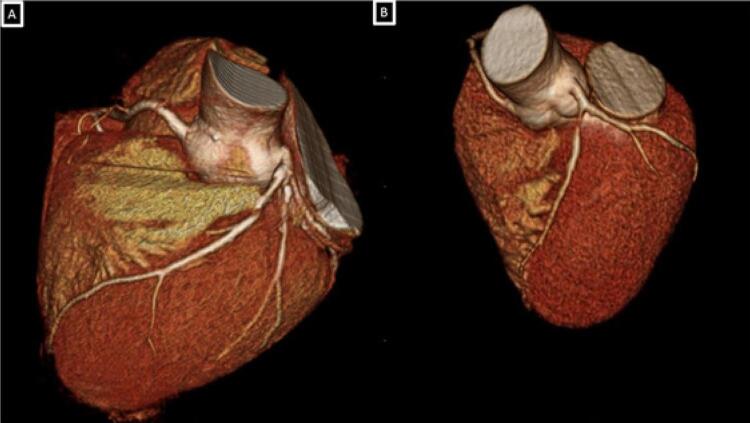
Reconstrução tridimensional (volume rendering) demonstrando importante redução luminal segmento proximal da artéria descendente anterior em ambos os pacientes (Paciente 1 - A / Paciente 2 - B).

**Figura 3:**
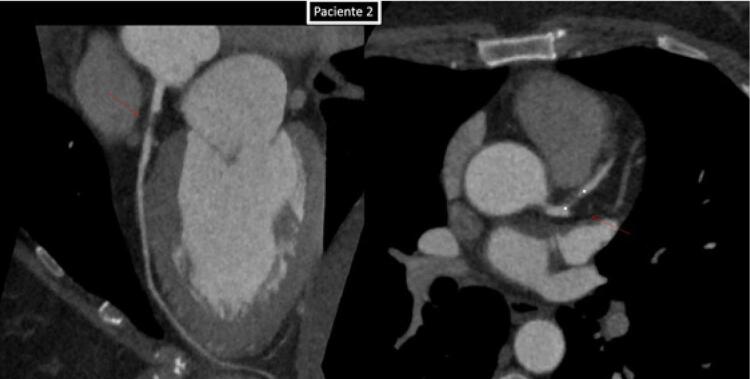
Angiotomografia das artérias coronárias. Imagem da Esquerda - reconstrução curva evidenciando placa mista no segmento proximal da artéria descendente (setas vermelhas), determinando acentuada redução luminal. Imagem da direita - imagem no plano axial do segmento proximal da artéria descendente anterior, na topografia da lesão (seta vermelha), demonstrando redução luminal crítica no paciente 1.

**Figura 4:**
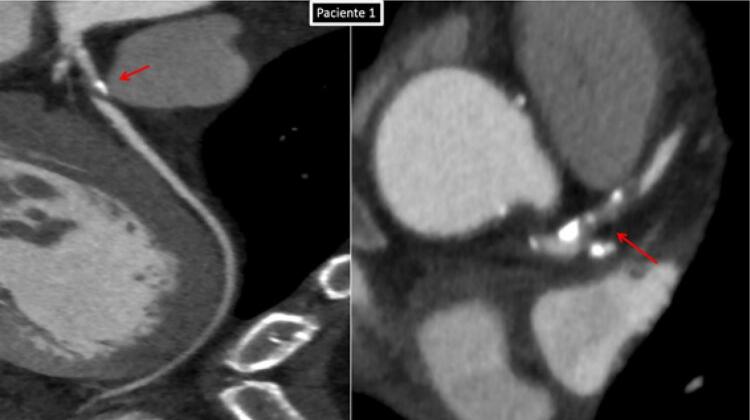
Angiotomografia das artérias coronárias. Imagem da Esquerda - reconstrução curva evidenciando placa mista no segmento proximal da artéria descendente (setas vermelhas), determinando acentuada redução luminal. Imagem da direita - imagem no plano axial do segmento proximal da artéria descendente anterior, na topografia da lesão (seta vermelha), demonstrando redução luminal crítica no paciente 2.

No nosso serviço, ao realizarmos a investigação de dois pacientes: paciente (1) masculino, tabagista, com queixa de dor atípica em repouso intermitente (CCS-IV) que após realização da angiotomografia de artérias coronárias como primeiro teste diagnóstico, apresentou episódio de dor, sendo encaminhado para a realização de eletrocardiograma de 12 derivações que demonstrou padrão A da Síndrome Wellens. Paciente (2) feminina, angina CCS2, com história familiar positiva (mãe infartou aos 35 anos) vem com eletrocardiograma evidenciando o padrão B de Wellens (Figura 1, 1A e 1B), angiotomografia confirmando os mesmos achados do paciente 1. Ambas imagens nas angiotomografias evidenciam placa segmentar, com sinais de vulnerabilidade determinando obstrução proximal importante do segmento proximal da artéria descendente anterior, prontamente à leitura (Figuras 2, 3, 4 e 5). A placa com características de vulnerabilidade apresentava-se parcialmente calcificada, com grande volume, remodelamento positivo e baixa atenuação.

**Figura 5:**
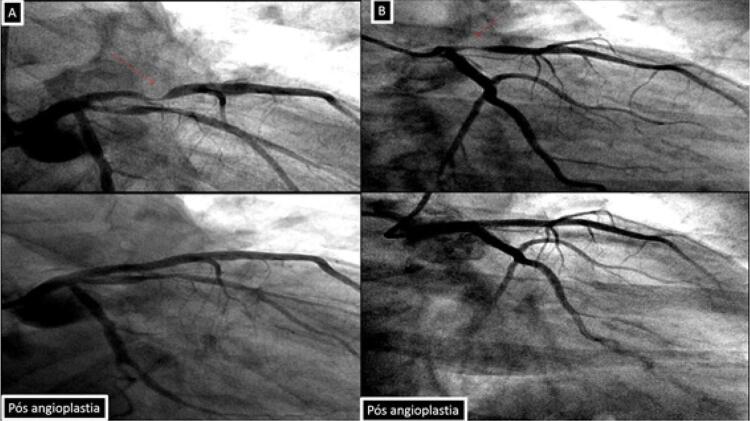
Cineangiocoronariografia. Imagem acima – lesão crítica no segmento proximal da artéria descendente, confirmando os achados tomográficos. Imagem abaixo – imagem após tratamento demonstrando recanalização efetiva da lesão. (Paciente 1 - A / Paciente 2 - B)

Após encaminhamento ao setor de emergência os pacientes obtiveram confirmação à cinengiocoronariografia sendo realizada angioplastia com sucesso da artéria descendente anterior (Figura 6).

Até onde sabemos, esse foi o primeiro relato da correlação eletrocardiograma – angiotomografia para a Síndrome de Wellens.
