# Supplementary material for: Correlação Angiotomográfica-Eletrocardiográfica na Síndrome de Wellens
Source: Arq Bras Cardiol. 2021 Feb 19;116(2):363–6. [Article in Portuguese] doi: 10.36660/abc.20200200 (PMC7909960; doi:10.36660/abc.20200200)
Supplement: Translation

# Translation: Radiologic-Electrocardiography Correlation in Wellens Syndrome

**Authors:** Eduardo Kaiser Ururahy Nunes Fonseca, Nevelton Heringer, Marcelo L. Montemor, Luiz Francisco Rodrigues de Ávila, Carlos Eduardo Rochitte

**Affiliations:** 1 BrasilUniversidade de São Paulo Faculdade de Medicina Hospital das Clínicas Instituto do Coração, São Paulo, SP - Brasil

**Keywords:** Electrocardiography, Coronary Vessels, Computed Tomography Angiography, Myocardial Infarction, Coronary Angiography

Wellens Syndrome,^1^ also known as “Anterior Descending Coronary T-wave syndrome”, was described in 1982 by Dr. Henrick Joan Joost (Hein) Wellens, a Dutch physician who also contributed to the characterization of the reentry mechanism in Wolff–Parkinson–White syndrome (WPWS).

Originally described during hospital admission (60% at admission and 40% at the follow-up) of patients with unstable angina, it was characterized by the occurrence of 2 electrocardiographic patterns, with pattern A in 25% of patients and B in 75% of patients.

Pattern A shows the occurrence of biphasic T-wave in leads V2 and V3 and can be found from V1 to V6, whereas pattern B shows inverted and symmetrical T-wave in V2 and V3, with both patterns occurring without the association of Q-waves or pathological QS complexes, with normal R-wave progression and without evidence of ventricular hypertrophy.

These electrocardiographic findings are not very sensitive (69%) but are highly specific (89%)^2^ for important obstructive disease in the proximal segment of the anterior descending coronary artery, which if not properly addressed, can determine extensive anterior infarction and high risk of mortality.

Therefore, the performance of provocative ischemia tests is discouraged in the presence of electrocardiographic findings of Wellens Syndrome.^3^

In our service, we conducted the investigation of two patients: patient (1) male gender, smoker, complaining of intermittent atypical pain at rest (CCS-IV) who, after undergoing a coronary artery angiotomography as the first diagnostic test, had an episode of pain, being referred to a 12-lead electrocardiogram that showed pattern A of Wellens Syndrome. Female patient (2), with CCS2 angina, with a positive family history (mother had an infarction at 35 years old) came with an electrocardiogram showing pattern B of Wellens Syndrome (Figure 1, 1A and 1B), and the angiotomography confirmed the same findings as in patient 1. Both angiotomography images show segmental plaque, with signs of vulnerability determining significant proximal obstruction of the proximal segment of the anterior descending artery, promptly at the reading (Figures 2, 3, 4 and 5). The plaque with characteristics of vulnerability was partially calcified, showing a large volume, positive remodeling and low attenuation.

**Figure 1:** – ECG images of both patients, showing the patterns of Wellens syndrome (Patient 1 - A / Patient 2 - B).

**Figure 2:** – Three-dimensional reconstruction (volume-rendering technique) showing important luminal reduction in the proximal segment of the anterior descending artery in both patients (Patient 1 - A / Patient 2 - B).

**Figure 4:** Angiotomography of the coronary arteries. Left image - curved reconstruction showing mixed plaque in the proximal segment of the descending artery (red arrows), resulting in marked luminal reduction. Right image - axial image of the proximal segment of the anterior descending artery, on the lesion topography (red arrow), showing critical luminal reduction in patient 2.

**Figure 3:** – Angiotomography of the coronary arteries. Left image - curved reconstruction showing mixed plaque in the proximal segment of the descending artery (red arrows), resulting in marked luminal reduction. Right image - axial image of the proximal segment of the anterior descending artery, on the lesion topography (red arrow), showing critical luminal reduction in patient 1.

**Figure 5:** Coronary angiography. Top image - critical lesion in the proximal segment of the descending artery, confirming the tomographic findings. Bottom image – post-treatment image showing effective recanalization of the lesion. (Patient 1 - A / Patient 2 - B)

After being referred to the emergency department, the patients’ condition was confirmed at the coronary angiography, and an anterior descending artery angioplasty was successfully performed (Figure 6).

To the best of our knowledge, this is the first report of an electrocardiogram-angiotomography correlation for Wellens Syndrome.
